# Distribution of Bone-Marrow-Derived Endothelial and Immune Cells in a Murine Colitis-Associated Colorectal Cancer Model

**DOI:** 10.1371/journal.pone.0073666

**Published:** 2013-09-10

**Authors:** Chuan-Xing Xiao, Huan-Huan Wang, Ying Shi, Ping Li, Yun-Peng Liu, Jian-Lin Ren, Bayasi Guleng

**Affiliations:** 1 Department of Gastroenterology, Zhongshan Hospital affiliated to Xiamen University, Xiamen, Fujian Province, China; 2 Faculty of Clinical Medicine, Medical College of Xiamen University, Xiamen, Fujian Province, China; University College London, United Kingdom

## Abstract

Inflammatory bowel disease (IBD) can lead to an increased risk of developing colorectal cancer (CRC). The aim of this study was to establish a model for combined bone marrow transplantation (BMT) and colitis-associated colorectal cancer (CAC) and to define the contribution of BM-derived cells during the inflammation associated with carcinogenesis. We established a model for BMT using green fluorescent protein (GFP) transgenic mice, followed by AOM/DSS-induced CAC, and performed confocal microscopy analysis on *in vivo* living tissue and frozen tumor sections. Our imaging analyses showed that GFP-positive cells extensively infiltrated the tumor stroma and that some WGA and GFP or CD31 and GFP double-positive cells were observed in the lining of tumor vessels. Flow cytometry analysis of the tumor-infiltrating cells showed that the GFP-positive CD11c+ DCs cells were one-third of the GFP+/CD11C- cells, and that half of these DCs (0.96% vs 1.02%) were GFP-positive BM-derived cells. The majority of CD4^+^ T cells were GFP-negative (12.02% vs 1.9%), and we discovered a novel CD4^+^ CD11c^+^ DC subset (0.34% vs 1.64%). In conclusion, we defined the distribution of BM-derived endothelial cells, CD11c^+^ DCs and CD4^+^ T cells in tumors. This model might be useful for elucidating the diverse BM-derived cell types and functions during the progression of colitis-associated colorectal cancer.

## Introduction

Colorectal cancer (CRC) is one of the leading causes of cancer-related death in the western hemisphere. Its development occurs sporadically or can be a long-term complication of chronic inflammation, as seen in inflammatory bowel disease (IBD) [1]. The cumulative risk for acquiring CRC can increase to approximately 20% in patients with IBD who live for 30 years with the disease [2,3]. Colitis-associated colon cancers develop in chronically inflamed mucosa and are believed to develop in a stepwise manner, with the inflamed mucosa giving rise to dysplasia and ultimately to cancer. Chronic inflammation is believed to promote carcinogenesis. Clinical studies have shown that patients with colitis have a 2- to 8-fold relative risk of developing colorectal cancer compared to the general population [4].

Mice given DSS to induce inflammation (AOM/DSS) show an increased incidence and extent of colon tumor formation compared with wild-type mice [5]. Individual components of the innate and adaptive immune responses have also been implicated in carcinogenesis [4]. Proinflammatory factors from the innate and adaptive immune systems contribute to the development and growth of colon neoplasia. Bone marrow-derived progenitor cells, which contribute to the inflammatory response and tumor neovascularization, have been suggested to have diverse roles in tumor progression. However, little is known about the role of bone marrow-derived cells in colitis-associated colon cancers (CAC) [6].

Bone marrow-derived cells are surprisingly plastic [7], sometimes assuming functions outside the hematopoietic system. In bone marrow transplantation (BMT) recipients, investigators have found donor-derived cells in diverse non-hematopoietic tissues such as skeletal muscle, cardiac muscle, vascular endothelium, neuronal cells and epithelial cells [8]. Bone marrow-derived cells have also been shown to play multiple roles in tumor development. Bone marrow-derived endothelial progenitor cells (EPCs) contribute to tumor neovascularization, whereas cells of the immature myeloid lineage that have diverse roles in tumor progression as cancer-initiating cells act as tumor-associated fibroblasts that orchestrate the desmoplastic reaction [9].

Convincing evidence supports the notion that bone marrow-derived cells play an important role in regulating tumor angiogenesis and progression [10–12] and may contribute to tumor angiogenesis by secreting pro-angiogenic factors or by direct incorporation into the tumor vasculature [13,14]. Circulating EPCs mobilized from the bone marrow have been detected in the peripheral blood of several species and have been shown to be involved in neoangiogenesis in tumors as well as in the formation of new vessels after trauma, burn injury, and myocardial infarction [15–17]. Moreover, we have previously documented that murine bone marrow-derived CXCR4-positive progenitor cells contribute to tumor growth by promoting tumor angiogenesis [18].

However, accumulating evidence indicates that bone marrow-derived progenitor cells have diverse roles in inflammatory responses during tumor progression. Bone marrow-derived inflammatory cells in the tumor microenvironment promote tumor angiogenesis, tumor proliferation, survival and invasion and suppress the specific anti-tumor immune response of CD4 and CD8 lymphocytes [19]. Thus, it is important to determine the relationship between bone marrow-derived cells and specific immune cells, cytokines, and chemokines during carcinogenesis. All lymphocytes originate within the bone marrow from a common lymphoid progenitor before differentiating into their distinct lymphocyte types. B cells mature into B lymphocytes in the bone marrow, whereas T cells migrate to and mature in the thymus. Following maturation, the lymphocytes enter the circulation and peripheral lymphoid organs, where they search for invading pathogens and tumor cells [20]. Other types of cells, including dendritic cells (DC), neutrophils, and eosinophils, have also been reported to promote tumor angiogenesis [13,21]. DCs are considered to play an important role in tumor immuno-surveillance by eliciting tumor-specific T cells responses. Recent evidence indicates that DCs might also contribute to tumor angiogenesis by releasing proangiogenic factors, such as VEGF and IL-8, in response to hypoxia [22].

These findings, together with the intrinsic plasticity of bone marrow-derived cells and inflammatory cells in response to microenvironmental cues and the persistence of polarizing signals in the tumor microenvironment, suggest that bone marrow-derived cells within the tumor microenvironment might eventually produce pro-angiogenic and pro-invasive factors that promote tumor progression [19].

In this study, we established combined BM transplantation and a colitis-associated colorectal cancer model to define the distribution of BM-derived endothelial cells, CD11c^+^ DC and CD4^+^ T cells in tumors. This model provides a powerful tool for studying the contributions of diverse BM-derived cells during colitis-associated colorectal cancer progression and may reveal novel BM-derived cell types and functions that could be applicable in future therapeutic strategies.

## Materials and Methods

### Animals and cells

C57BL/6, BALB/c and GFP mice were purchased from the laboratory animal center, Shanghai, China. All procedures involving experimental animals were performed in accordance with protocols approved by the Committee for Animal Research of the University of Xiamen and complied with the Guide for the Care and Use of Laboratory Animals (NIH publication No. 86-23, revised 1985). Mice were maintained in a specific pathogen-free (including *Helicobacter* -free and parvovirus-free) environment and were generally used between 6 and 8 weeks of age. CT26-WT and CMT93 cells were derived from BALB/c and C57BL/6 mice, respectively. CT26-WT and CMT93 cells (commercially purchased from ATCC, USA) were cultured in DMEM and supplemented with 10% fetal bovine serum, 100 U/ml penicillin, 100 mg/ml streptomycin, 2 mM glutamine, 1 mM sodium pyruvate and 0.1 mM non-essential amino acids at 37°C and 5% CO2.

### Bone marrow transplantation

The bone marrow of lethally irradiated C57BL/6 mice was reconstituted by transplantation with bone marrow cells from GFP chimeric mice (GFP-BMT mice). Briefly, wild-type C57BL/6 mice were lethally irradiated with a dose of 950 rads, after which 2x10^6^ bone marrow cells from GFP mice were injected into the tail veins of the irradiated recipient mice. The bone marrow cells from the GFP-BMT mice were evaluated at 4 weeks after bone marrow transplantation, and the degree of chimerism was measured by flow cytometry. More than 90% of the cells in the circulating mononuclear cells were replaced by GFP-positive cells under these experimental conditions.

### Colitis-associated colorectal cancer (CAC) and tumor implantation model

The GFP-BMT mice were injected i.p. with 10 mg/kg of AOM in 0.2 ml saline. One week after AOM administration, 3% DSS was administered in the drinking water for 7 days and then switched to normal drinking water for 14 days. This procedure was repeated for 3 cycles, followed by a switch to normal water. Tumors were induced by s.c. injection of 2x10^6^ CT26-WT and CMT93 cells into the flank at 4 weeks after bone marrow transplantation. Large tumors were typically observed by 4 weeks after tumor implantation, and the tumor tissues were harvested for histologic analysis.

### Confocal microscopy

In GFP-BMT CAC mice, blood vessels were stained with 50 µg Alexa Fluor® 647 WGA (Molecular Probes, InvitroGen), which was injected retrorbitally. Seven minutes after Alexa Fluor® 647 WGA administration, mice were euthanized by CO2 asphyxiation. All procedures were performed under anesthesia (0.5 mL Avertin, i.p.). Tissues were immediately removed, opened by longitudinal incision and rinsed with PBS. Living tumor tissue specimens were imaged with a Bio-Rad Radiance 2000 confocal microscope (Bio-Rad, Hercules, CA). Image acquisition was carried out with Laser Sharp Scanning Software (Bio-Rad), and 3D reconstructions were completed with Volocity software (Waltham, MA).

### Flow cytometry analysis

Tumor tissue was isolated from GFP-BMT CAC mice and cut into pieces in 1 mg/ml of collagenase Ⅳ. Collagenase digestion was performed in collagenase solution for 45 minutes at 37°C. Eluted cells were then passed through a 70-µl cell strainer. The cells were spun down for 10 minutes at 1400 rpm at room temperature and collected. The cells then were incubated in 10% donkey serum and Fc blocked (BD Pharmingen, San Jose, CA) for 20 minutes at 4°C, after which they were stained with fluorescent conjugated antibodies. APC-conjugated anti-mouse CD11c (HL-3) and PE-conjugated anti-mouse CD4 (OX-38) were purchased from BD Pharmingen (San Diego, CA) and used for FACS analysis. The cells were analyzed on a FACSCaliber cytometer (BD Bioscience, San Diego, CA) and then analyzed using FlowJo software (Tree Star, Ashland, OR). 

### Immunofluorescence staining

C57BL/6 mice were euthanized by CO_2_ asphyxiation, and the colon tissues were immediately removed, fixed for 4 to 5 hours at room temperature in GFP fixation buffer, embedded in Tissue-Tek OCT compound (Miles Inc., Elkhart, IN), and snap frozen and stored at -80°C, after which 5-10 µm sections were cut on a cryostat and transferred to glass microscope slides pre-coated in poly-lysine (Sigma-Aldrich). Sections were then fixed with cold acetone for 20 minutes at room temperature. Fixed sections were washed three times with PBS and incubated with 5% normal donkey serum and 0.02% BSA-PBS for one hour at room temperature. The primary antibodies used were specific to CD31 (initial concentration, 0.5 mg/ml; dilution, 1/500; eBioscience), CD4 (dilution, 1/200; BD), CD11c (dilution, 1/1000; BD), and Ly6C (dilution, 1/100; BD). DAPI (Vector Laboratories, Burlingame, CA) was used for nuclear staining.

### Statistical analysis

The data are expressed as the mean ± SD. Comparisons between groups were analyzed using Student’s *t*-test. *P* < 0.05 was considered statistically significant.

## Results

### Establishing a combination of bone marrow transplantation and colitis-associated colorectal cancer model

**Figure 1:**
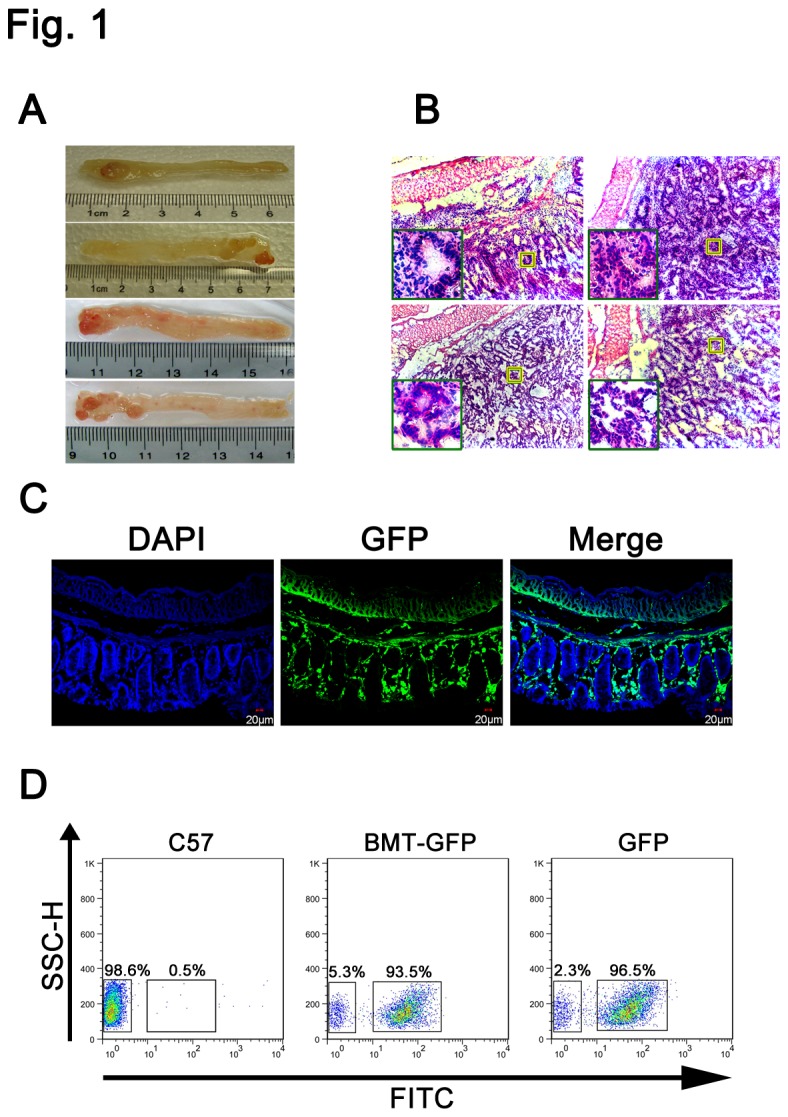
The combined bone marrow transplantation and colitis-associated colorectal cancer model. (A) Macroscopic view shows a number of colonic tumors in the distal colons of the mice model. (B) H&E stains of serial sections of mouse colon tissues. (C) Confocal microscopy analysis of frozen colon sections showed the bone marrow-derived cells (green) in the colon. (D) Flow cytometry analysis of peripheral mononuclear cells in the GFP-BMT model.

Wild-type C57BL/6 mice were lethally irradiated with a total dose of 950 rads, after which 2x10^6^ bone marrow cells from GFP mice were injected into the tail veins of the irradiated recipient mice. Four weeks after bone marrow transplantation, colon tissue was isolated from GFP-BMT mice for confocal microscopy analysis, and the degree of chimerism was measured by flow cytometry. We found that the intestine was filled with green fluorescence, and more than 90% of the circulating mononuclear cells in the recipient mice were GFP-positive (Figure 1C, D).

The GFP-BMT mice were then injected i.p. with 10 mg/kg AOM in 0.2 ml saline. One week after AOM administration, 3% DSS was administered in the drinking water for 7 days, followed by 14 days of normal water; this alternation was repeated for 3 cycles. We observed intestinal tumors in the majority of mice, and the tumor types were confirmed by histological analysis (Figure 1A, B).

### Bone-marrow-derived endothelial cells contributed to angiogenesis during colitis-associated colorectal cancer

**Figure 2:**
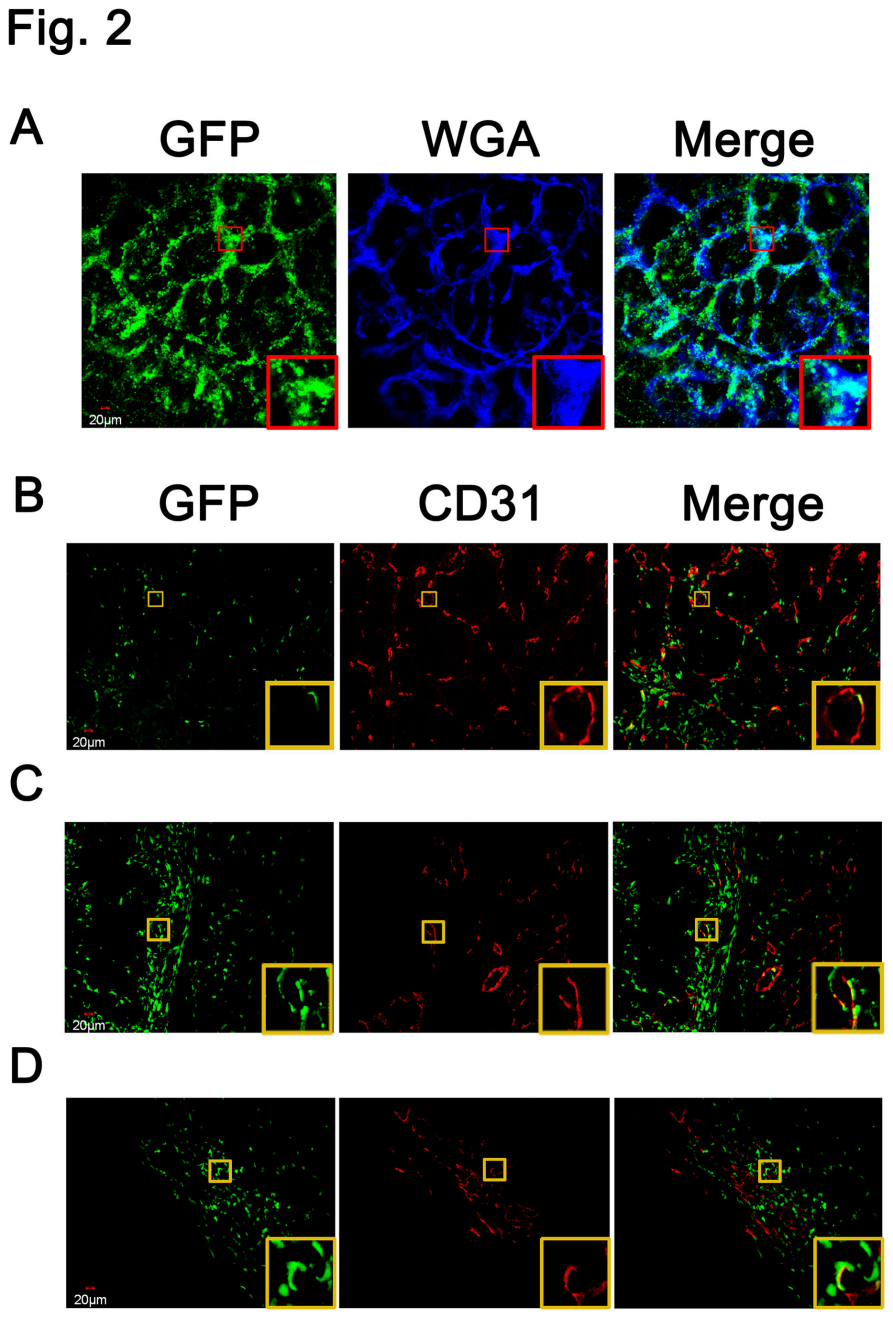
Bone marrow-derived endothelial cells contributed to colitis-associated colon cancer angiogenesis. (A) Blood vessels were stained with 647 WGA (blue), which was injected retrorbitally. Confocal microscopy analysis from living tissue specimens showed that the BM-derived cells (green) infiltrated the stroma, and a portion of cells were hemmed by a WGA-647-positive (blue) margin at the tumor endothelium. Double-positive cells were observed lining the vessels. (B) The frozen tumors sections were stained with an anti-CD31 antibody to detect BM-derived endothelial cells (green). GFP-positive cells (green) with CD31-positive margins were seen in the tumor endothelium. C (CMT93) and D (CT26) xenograft tumor tissues isolated from BM transplantation mice were stained with anti-CD31 (red) antibodies. GFP-positive cells were observed around the tumor vessels, and some lined the endothelium.

To investigate whether BM-derived endothelial cells contributed to tumor angiogenesis in this model, we analyzed the expression of GFP in the endothelium of tumors in GFP-BMT mice following the induction of colitis-associated colon cancer. Confocal microscopy analysis on living tissue specimens showed that the GFP-positive cells extensively infiltrated to the tumor stroma, and some of the cells were hemmed by a WGA-positive margin at the tumor endothelium. WGA and GFP double-positive cells were observed lining the vessels (Figure 2A). Frozen tumor sections were stained with anti-CD31 antibody (n=5), and as shown in Figure 2B, GFP-positive cells infiltrated the tumor endothelium. Taken together, our data indicated that BM-derived endothelial cells contributed to colitis-associated colon cancer angiogenesis.

Additionally, consistent with our previous report (22), we detected GFP-positive endothelium in CMT93 and CT26 xenograft tumors in GFP-BMT mice (Figure 2C, D).

### Distribution of bone-marrow-derived CD11c^+^ dendritic cells and CD4^+^ T cells in colitis-associated colorectal cancer

**Figure 3:**
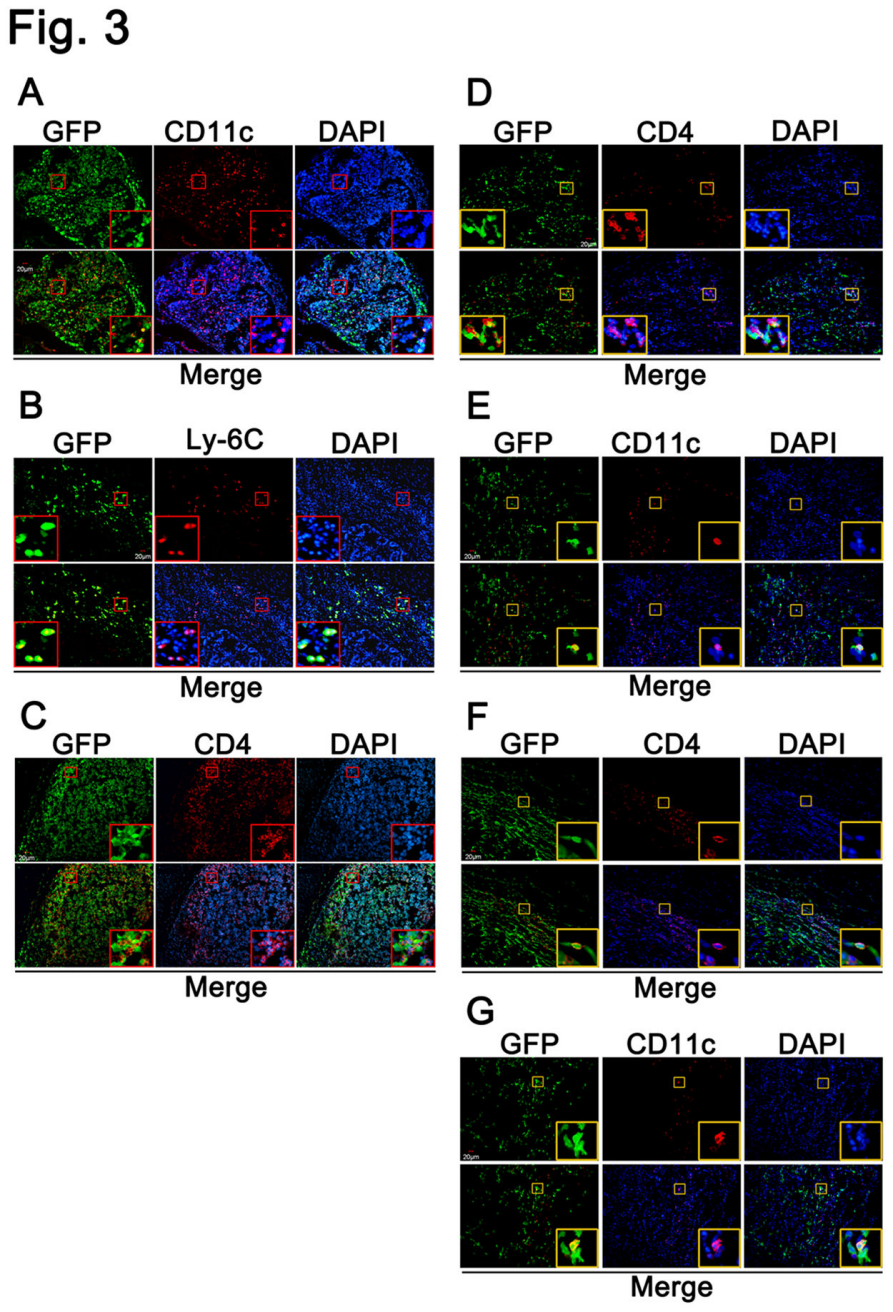
Confocal microscopy analysis of the distribution of BM-derived inflammatory cells in the tumor microenvironment. (A–C) Immunofluorescent staining of the combined BMT and colitis-associated colorectal cancer samples with anti-CD11c, Ly-6C and CD4-specific antibodies (red). (D–E) CMT93 tumor tissues isolated from BM transplantation mice were stained with anti-CD11c and CD4-specific antibody (red). (F–G) CT26 tumor tissues isolated from BM-transplanted mice were stained with anti-CD11c and CD4-specific antibody (red).

To determine the distribution of BM-derived inflammatory cells in the tumor microenvironment, we analyzed the mice that underwent bone marrow transplantation followed by the induction of colitis-associated colorectal cancer by confocal microscopy. As show in Figure 3A, B and C, the tumor tissues were extensively infiltrated by CD11c^+^, Ly-6C and CD4^+^ immune cells, most of which expressed GFP. In addition, we detected the GFP-positive CD11c+ DCs and CD4+ T cells in the tumor of the CMT93 and CT26 xenograft tumors in GFP-BMT mice (Figure 3D, E, F and G).

**Figure 4:**
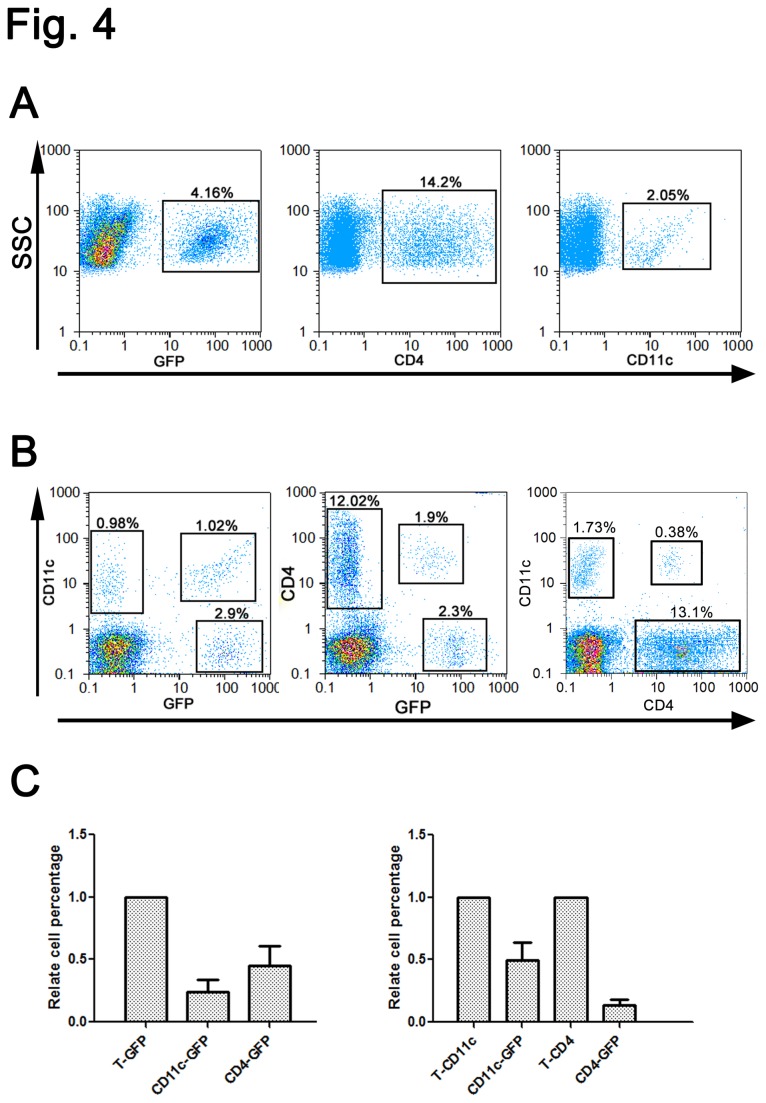
Flow cytometry quantitative analysis of the distribution of BM-derived CD11c^+^ DC and CD4^+^ T cells in colitis-associated colorectal cancer. (A) Isolation of cells from tumor tissue followed by FACS analysis showed the proportion of the bone marrow-derived cells, CD4^+^ cells and CD11c^+^ DCs in the colon tumor tissue. (B–C) Composition of the CD11c^+^ and CD4^+^ T cells derived from bone marrow. (C) Bars represent the mean ± SD; sample analyses were conducted in triplicate.

To quantitatively define the distribution of BM-derived CD11c+ DC and CD4+ T cells in our colitis-associated colorectal cancer model, we isolated the tumor-infiltrating cells from the mouse tumor tissues and performed FACS analysis. Our isolation protocol data indicated that among tumor-infiltrating cells, 4.16 ± 0.12% were GFP-positive cells, 14.2 ± 0.34% were CD4+ cells, and 2.05 ± 0.23% were CD11c+ DCs (Figure 4A). Surprisingly, the GFP-positive CD11c+ DCs cells were one-third of the GFP+/CD11C- cells, and half of the DCs (0.96% vs 1.02%) were GFP-positive bone marrow original cells. The majority of CD4+ T cells were GFP-negative (12.02% vs 1.9%). Interestingly, we found a new CD4+ CD11c+ DC subset (0.34% vs 1.64%) in this model (Figure 4B, C).

## Discussion

Inflammatory bowel disease (IBD) can lead to an increased risk of developing colorectal cancer (CRC). The molecular pathogenesis of IBD-associated colorectal cancer differs from that of sporadic CRC [23]. Inflammation is a key contributor to carcinogenesis, and chronic inflammatory diseases such as IBD initiate complex pathways to neoplasia [24]. In this study, we used a potent carcinogenic agent, AOM, that induces DNA adducts via alkylating species. These adducts form covalent bonds between the reactive carcinogen and the genomic DNA, resulting in mutations during DNA repair and promoting tumor development. Such mutations include murine genetic mutations in tumorigenesis pathways (APC, p53, Msh2) [25]. Long-term administration or repeated cycles of DSS, a chemical that causes epithelial injury, can induce chronic colitis and subsequent dysplasia in rodents [26].

Over the past decade, remarkable advances have been made in the understanding of stem cell biology and the role of stem cells in diseases such as CRC [27]. In particular, discoveries related to the control of stem cell proliferation and to how the dysregulation of proliferation leads to oncogenesis have been at the forefront. Thus, we established a combined bone marrow transplantation and colitis-associated colorectal cancer model to define the distribution and function of BM-derived cells in colon cancer.

Recently, circulating EPCs mobilized from the BM have been detected in the peripheral blood of several species and have been shown to be involved in neoangiogenesis in tumors as well as in the formation of new vessels after trauma, burn injury, and myocardial infarction [28,29]. Most solid tumors require a vascular supply to provide oxygen and nutrients to enhance tumor progression and invasion. The tumor can either exploit existing vessels, or recruit and mobilize BM-Derived cell to induce neovascularization [30]. EPCs originate from BM-Derived cell and possess the capacity to differentiate into mature endothelial cells, contributing to the complex process of tumor neovascularization. There is evidence that tumor angiogenesis can be stimulated by tumor cell secreted CXC chemokine ligands CXCL5 and CXCL8, via their common receptor CXCR2 [31]. Audollent et al show that Bone marrow-derived endothelial and hematopoietic precursors cells enhance the metastasis of colon cancer in an orthotopic nude mice model [32]. Our previous report showed that BM-derived endothelial cells were detected in xenograft tumors using Colon38 and Panco2 cells [18]. In this study, we showed via confocal microscopy analysis that BM-derived endothelial cells participated in colitis-associated colon cancer angiogenesis. Furthermore, we detected the expression of GFP in the endothelium of CMT93 and CT26 tumor xenografts in GFP-BMT mice, consistent with our previous report [18].

Similar to other solid malignancies, colorectal and colitis-associated tumors are infiltrated by various types of immune cells [33]. Cells of the innate immune system, such as neutrophils, mast cells, natural killer (NK) cells, dendritic cells, and tumor-associated macrophages, can be easily detected in these tumors. In addition, advanced tumors recruit specific myeloid subsets that represent a phenotypically heterogeneous but functionally similar population of CD11b^+^ Gr1^+^ cells, termed myeloid-derived suppressor cells [34,35]. These cells share some characteristics with monocytes, macrophages, neutrophils, and DCs and help suppress antitumor immune responses and tumor angiogenesis. Previous studies have established that BM-derived APCs are primarily responsible for the induction of tumor-induced T cell tolerance [36]. BM-derived DC, classical marker of CD11c, can capture and present peripheral tissue-specific antigens to naïve CD8^+^T cells, leading to their deletion [37]. There is a similar requirement for the processing of parenchymal self antigen by host DC in the induction of CD4^+^T cell tolerance in tumor. BM-derived DCs from BALB/c mice are able to promote expansion of CD4^+^CD25^+^Foxp3^+^ T cells *in vitro* and that this occurs independent of the maturation state of the DCs as mature and immature cells are capable of expanding the Foxp3^+^cell population [38,39]. This immune ignorance and the immune tolerance mechanisms contribute to tumor development. We showed the distribution of BM-derived inflammatory cells (CD11c^+^, Ly-6C and CD4^+^ T cells) in the tumor microenvironment. Surprisingly, the GFP-positive CD11c+ DCs cells were one-third of the GFP+/CD11C- cells, and half of all DCs were GFP-positive BM original cells. We also discovered a new CD4^+^ CD11c^+^ DC subset (0.34 vs 1.64) in our colitis-associated colorectal cancer model. Cells of the adaptive immune system are recruited into colorectal and colitis-associated tumors, where they play either pro- or antitumorigenic roles [40]. T cells, for instance, are required not only for inflammation, cancer development, and tumor progression but also for anticancer immunity [41].

We successfully established a combined BM transplantation and colitis-associated colorectal cancer model. This model provides a powerful tool for studying the contributions of diverse BM-derived cells during the progression of colitis-associated colorectal cancer and may reveal novel BM-derived cell types and functions that could be applicable in future therapeutic strategies.
